# Evolution and diversity of secretome genes in the apicomplexan parasite *Theileria annulata*

**DOI:** 10.1186/1471-2164-11-42

**Published:** 2010-01-18

**Authors:** William Weir, Tülin Karagenç, Margaret Baird, Andy Tait, Brian R Shiels

**Affiliations:** 1 Division of Veterinary Infection and Immunity, University of Glasgow, Faculty of Veterinary Medicine, Institute of Comparative Medicine, Bearsden Road, Glasgow, Scotland, G61 1QH, UK; 2 Department of Parasitology, Adnan Menderes University, Faculty of Veterinary Medicine, Batı Kampus, Işıklı, Aydın, Turkey

## Abstract

**Background:**

Little is known about how apicomplexan parasites have evolved to infect different host species and cell types. *Theileria annulata *and *Theileria parva *invade and transform bovine leukocytes but each species favours a different host cell lineage. Parasite-encoded proteins secreted from the intracellular macroschizont stage within the leukocyte represent a critical interface between host and pathogen systems. Genome sequencing has revealed that several *Theileria*-specific gene families encoding secreted proteins are positively selected at the inter-species level, indicating diversification between the species. We extend this analysis to the intra-species level, focusing on allelic diversity of two major secretome families. These families represent a well-characterised group of genes implicated in control of the host cell phenotype and a gene family of unknown function. To gain further insight into their evolution and function, this study investigates whether representative genes of these two families are diversifying or constrained within the *T. annulata *population.

**Results:**

Strong evidence is provided that the sub-telomerically encoded *SVSP *family and the host-nucleus targeted *TashAT *family have evolved under contrasting pressures within natural *T. annulata *populations. *SVSP *genes were found to possess atypical codon usage and be evolving neutrally, with high levels of nucleotide substitutions and multiple indels. No evidence of geographical sub-structuring of allelic sequences was found. In contrast, *TashAT *family genes, implicated in control of host cell gene expression, are strongly conserved at the protein level and geographically sub-structured allelic sequences were identified among Tunisian and Turkish isolates. Although different copy numbers of DNA binding motifs were identified in alleles of TashAT proteins, motif periodicity was strongly maintained, implying conserved functional activity of these sites.

**Conclusions:**

This analysis provides evidence that two distinct secretome genes families have evolved under contrasting selective pressures. The data supports current hypotheses regarding the biological role of TashAT family proteins in the management of host cell phenotype that may have evolved to allow adaptation of *T. annulata *to a specific host cell lineage. We provide new evidence of extensive allelic diversity in representative members of the enigmatic SVSP gene family, which supports a putative role for the encoded products in subversion of the host immune response.

## Background

Apicomplexan parasites are major pathogens of humans and domesticated animals. Infection of the mammalian host requires establishment within a range of different host cell types that can vary in a species-specific manner. While the molecular mechanisms these parasites use to manipulate the phenotype of the infected cell are beginning to be understood [1], little is known about how different parasite species within a genus have evolved to establish infection in cells of different lineages or novel host species. The genus *Theileria *encompasses species of tick-transmitted parasitic protozoa that infect domestic livestock and other mammals. Of the five species that cause clinical disease in cattle, the two most important are *T. annulata*, the agent of tropical theileriosis, which is widespread in North Africa, Southern Europe and Asia and *T. parva*, the agent of East Coast fever a highly fatal disease of cattle in East and Central Africa. Following inoculation by the tick vector, the parasite invades and transforms host leukocytes, which divide in synchrony with the intracellular multi-nucleate macroschizont stage. For *T. annulata*, parasite infection results in establishment of transformed myeloid cells but never T lymphocytes. For *T. parva*, T cells are the preferred host cell type and while macrophages can be infected they are not transformed [2]. Establishment of the transformed leukocyte is characterised by activation of a number of host cell transcription factors and the production of inflammatory cytokines, the profile of which varies considerably between *T. annulata *and *T. parva *infected cells [3]. In *T. annulata*, recovery from primary challenge results in non-sterile immunity, involving T cell recognition of class I presented parasite peptides, followed by the development of the persistent carrier state [4]. This state is highly important for transmission of the parasite and in *T. annulata *causes significant economic losses due to sub-clinical infection [5]. The mechanisms by which the parasite avoids clearance from the host are obscure although in part, it is believed that low-grade infection is maintained by macroschizont-infected leukocytes residing in immunologically privileged sites [4]. It is also likely that the macroschizont-infected cell actively evades and subverts the bovine immune response [2,3,6] but the molecular mechanisms involved have not been elucidated.

A comparative analysis of the genomes of *T. annulata *and *T. parva *has shown that genes encoding polypeptides predicted to be either on the surface of the merozoite (the extra-cellular, bloodstream stage) or secreted by the macroschizont exhibit relatively high inter-species ratios of non-synonymous to synonymous nucleotide substitutions (d_N_d_S_) [7]. For the macroschizont stage, this indicates that genes encoding secreted proteins are more likely to be under positive selection at an inter-species level than genes encoding non-secreted products. In many other pathogens, elevated d_N_d_S _ratios have been observed for antigen-encoding genes. In an early study, d_N_d_S _ratios of a large number of homologous sequences lodged in Genbank were calculated, identifying 17 groups of genes across a range of species as being under the influence of positive selection, nine of which encoded surface antigens of parasites and viruses [8]. It has been shown that significant allelic diversity of antigen genes with positive d_N_d_S _ratios occurs within pathogen species [9] and in the case of *T. annulata *merozoite surface antigens, positive selection for allelic diversity has been attributed to host immune selection [10]. However, it is unlikely that full-length *Theileria *proteins expressed by the macroschizont are directly exposed to the host immune response since no parasite-encoded products have been identified on the surface of the infected leukocyte, despite intensive investigation [11]. Thus the reasons for elevated d_N_d_S _and positive selection in macroschizont-expressed genes encoding secreted products remain to be determined.

A number of genus-specific gene families have been identified in the genomes of *T. annulata *and *T. parva*, several of which are predicted to encode products secreted by the macroschizont into the host cell compartment [7]. Together with a number of single-copy genes, these families encode the predicted *Theileria *secretome that is likely to represent a critical interface between host and parasite systems. The present study was designed to investigate parasite gene families encoding products that may play a role at this interface and are evolving under positive, diversifying selection. In this study we have conducted an analysis of allelic diversity of *T. annulata *secretome gene families in order to provide insight into their evolution and putative function. For example, genes that have evolved to allow adaptation to a particular biological niche, such as a cell lineage or host species, would be predicted to be conserved within a parasite species but show divergence between species that exploit different host backgrounds or cell types. Consequently, gene families involved in host adaptation would be predicted to stabilise within each species and this would be reflected in evidence of purifying selection operating at the allelic level. In contrast, a gene family representing multiple paralogous antigens may be predicted to show evidence of diversification at both the inter-species and intra-species level. To investigate this hypothesis, the published *Theileria *genomes were compared in order to identify two positively selected gene families with distinctive and contrasting bioinformatic signatures. This would allow representative genes to be identified and the broad pattern of diversity exhibited by each family to be characterised and interpreted.

## Results

### Identification of secretome families subject to inter-species diversifying selection

**Figure 1:**
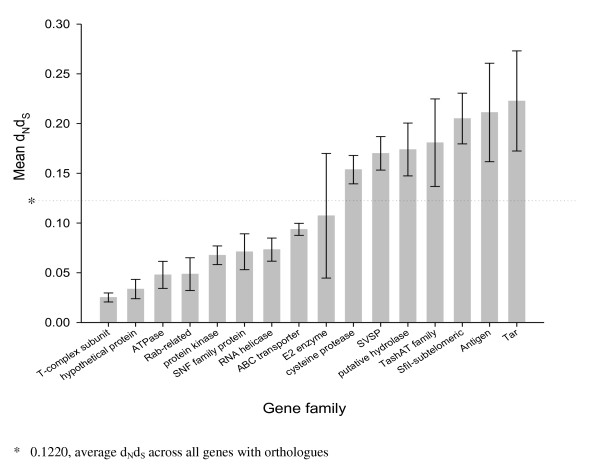
**Selection within *T. annulata*/*T. parva *gene families**. Fifteen gene families were identified in the genomes of *T. annulata *and *T. parva *containing seven or more members, together with a group of putative single-copy antigen-encoding genes (n = 10). The mean d_N_d_S _value along with the standard error for each of these families was calculated and the families ranked according to increasing mean d_N_d_S_. The low d_N_d_S _group consists of 'housekeeping' gene families and the high d_N_d_S _group includes several gene families which encode secreted products.

The ratios of non-synonymous to synonymous nucleotide substitutions (d_N_d_S _values) were determined for *T. annulata *families compared to their *T. parva *orthologues for all genes where an orthologous relationship had been determined and the mean value for each family computed. This analysis was performed using the 14 Tribe-MCL families that contain seven or more genes [7], together with two additional groups - genes annotated as antigens (such as *TaMS1*/*TpMS1 *[10] and *SPAG*/*p67 *[12]) and TashAT family genes [13,14]. Gene families were found to fall into two types - those with either below or above the average d_N_d_S _values for all genes (Figure 1). The nine families with below average d_N_d_S _consist of enzymes such as ATPases, protein kinases, RNA helicases and other housekeeping genes such as the ABC transporters, which are integral membrane proteins encoded in sub-telomeric regions. The variance of the d_N_d_S _value of these gene families is generally low, with the exception of the small E2 ubiquitin-conjugating enzyme family. Only two of the seven families with higher than average values encode products which belong to protein superfamilies present in other protozoan parasites, namely a family of cysteine proteases and a family of putative haloacid dehalogenase-like hydrolases. The remaining five groups of genes with higher than average d_N_d_S _values are *Theileria*-specific and comprise: (1) SVSP-encoding genes, (2) *TashAT *genes, (3) *SfiI sub-telomeric *genes, (4) known antigen-encoding genes and (5) *Tar/Tpr *genes. *SVSP *genes had the lowest variance, with almost all members exhibiting a high d_N_d_S _value while the two highest-ranking groups were the antigen-encoding genes and *Tar/Tpr *gene families, with mean d_N_d_S _values of 0.211 and 0.223 respectively. The known antigen-encoding genes include single-copy genes encoding GPI-anchored merozoite surface antigens which have been previously shown to be associated with elevated inter-species d_N_d_S _[7]. This group, therefore, cannot be classified as a major secretome gene family. A summary of the *Theileria*-specific secretome gene families, with higher than average d_N_d_S_, is shown in Table 1.

**Table 1:** *T. annulata/T. parva-*specific genes families with elevated d_N_d_S_

Family name	Number of genes in *T.a.*	Putative product	Multiple TMDs	Chromosomal location	EST data			Number of genes encoding a predicted signal sequence in *T.a.*	Predicted Function
					macro	mero	piroplasm		
*SVSP*	51	hypothetical proteins		sub-telomeric arrays	+	-	-	49	unknown
*TashAT*	17	host nuclear proteins		internal locus (Chr. II)	+	-	-	15	control of host cell phenotype
*SfiI sub-telomeric*	72	hypothetical proteins		sub-telomeric	+/-	-	-	26	unknown
*Tar/Tpr*	93	integral membrane proteins	√	internal loci	+/-	+/-	+/-	20	unknown

+ majority of members have associated EST hits, +/- minority of members have associated EST hits.

The TashAT family is a 17-member family which is present in *T. annulata *with an orthologous family of genes in *T. parva (TpHN) *that show a significant level of synteny [13-15]. The majority of TashAT-encoded proteins are either predicted to be or have been demonstrated to locate to the host nucleus [1,13,14]. In *T. annulata*, experimental evidence indicates that they are likely to function as modulators of host cell phenotype, possibly in concert with activated host cell transcription factors [13,16]. Typically, a TashAT protein comprises several domains with predicted functions including a signal peptide for secretion, nuclear localisation/DNA binding motifs, phosphorylation sites for the cell cycle dependent p34^cdc2 ^kinase (CDK) [17] and PEST motifs [13,14] enriched in proline (P), glutamic acid (E), serine (S) and threonine (T) that act as a signal for proteolytic degradation in a number of important regulatory proteins [18]. TashAT and TpHN proteins also contain a FAINT (Frequently Associated IN *Theileria*) domain, a *Theileria*-specific polymorphic domain of unknown function that is present in 166 predicted polypeptides in *T. annulata *and an equivalent number in *T. parva*, the majority of which posses a signal peptide [1,7]. Representative examples of PEST and FAINT domain distribution for TashAT and SVSP sequences can be viewed in [7] and in Additional file 1. Significant divergence has occurred between the *TashAT *and *TpHN *gene families [1,15], and this is most easily detected by the specific absence of AT-hook DNA binding/nuclear localisation motifs in *T. parva*. However, the majority of the orthologous pairs encode the same basic putative functional motifs, which occur in the same general order and are frequently located in an identical or similar position [1]. Together with phylogenetic analysis [15] this strongly indicates that expansion and divergence of *TashAT *and *TpHN *genes have occurred to allow functions specific for each species to evolve, a postulation which predicts that functional motifs specific to orthologues of one species would be conserved within that species. To investigate this hypothesis, members of the *TashAT *gene family were selected for analysis of sequence diversity across different parasite isolates.

To determine which of the other secretome gene families of *T. annulata *was most suitable to compare and contrast with the *TashAT *family, the SVSP-encoding genes, *SfiI sub-telomeric *genes and *Tar/Tpr *genes were bioinformatically screened to determine in which families the majority of proteins are predicted to be components of the macroschizont secretome. The *Tar/Tpr *family was considered for a similar analysis of selection but for the reasons outlined below was not considered as suitable for such analysis. This is the largest gene family in *T. annulata*, encoding 93 hypothetical proteins in the genome. Using available algorithms, only 20 *Tar/Tpr *members were found to encode polypeptides with a predicted signal sequence (11 of which are predicted to be membrane anchored) while a large proportion of family members encode multiple trans-membrane domains. Therefore, the available data suggests that many *Tar/Tpr *genes encode integral membrane proteins and it can be postulated that the majority of members are unlikely to contribute to the secretome. This observation coupled with current data indicating expression across multiple life-cycle stages and the possibility that d_N_d_S _values represent a degree of misalignment between orthologous genes meant that this gene family was not selected for comparison with the TashAT family.

### Sub-telomerically-encoded gene families

**Figure 2:**
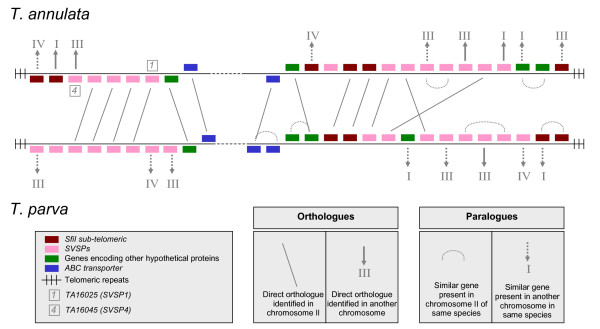
**Sub-telomeric regions of chromosome II of *T. annulata *and *T. parva***. Synteny is partially conserved between *T. annulata *and *T. parva *at sub-telomeric loci. For the majority of genes, direct orthologues were identified in each genome at the same locus (e.g. *SVSP1*) or at different loci (e.g. *SVSP4*) together with evidence of gene duplication and chromosomal recombination within each species. Chromosome numbers are shown in roman numerals

**Figure 3:**
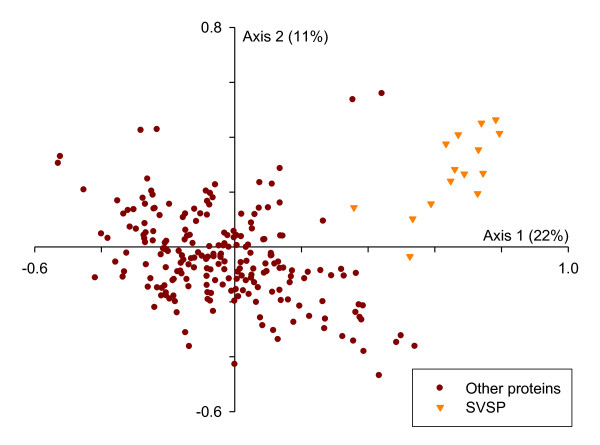
**Codon usage of the secretome**. Codon usage was analysed across the set of macroschizont expressed *T. annulata *genes encoding a signal peptide with an orthologue in *T. parva *(n = 218). Correspondence analysis was used to transform the data in order to explore variation in codon usage among these genes. 33% of the variation present in the dataset is illustrated on the graph as the first two principal components of the analysis and this clearly shows that SVSP-encoding genes display atypical codon usage.

SfiI sub-telomeric proteins are a large family of hypothetical proteins in *T. annulata *(n = 72). Only a subset of *SfiI *family members are expressed at the macroschizont stage and not all of these are predicted to be secreted [19]. In contrast, *SVSP *genes encode a family of Variable Sub-telomerically-encoded Secreted Proteins (SVSPs), of which almost all are expressed at the RNA level by the macroschizont stage of *T. annulata *and *T. parva *[1,7,20]. The 51 *SVSP *genes in *T. annulata *are distributed across every sub-telomere in short arrays, that are generally flanked by an ABC transporter centromerically and an *SfiI sub-telomeric *gene telomerically. Typically, *SVSP *genes comprise a single exon with the hypothetical product possessing a signal peptide at the N-terminus. Organelle-targeting motifs have not been identified in the majority of *T. annulata *SVSP proteins [19] suggesting they are secreted into the host cytosol. Direct orthologues, in *T. parva *have been identified for over half the family [7] and although nucleotide identity between orthologues ranges from 56% to 71%, mean amino acid identity is only 46%. To identify additional orthologous relationships, the amino acid sequences of *SfiI sub-telomeric *and *SVSP *genes in both species were aligned and clustered (data not shown). This process revealed a number of closely related sequence pairs from within each species, indicating paralogous genes probably arising from duplication events. For a number of genes, including most *SfiI sub-telomeric *genes, the closest neighbour was identified as a paralogue located in a different telomere, implying a high level of duplication and recombination within these families. A schematic representation of the gene organisation of a sub-telomere of chromosome II is depicted in Figure 2 and this organisation is typical of the other sub-telomeres. While both gene families display significant diversity within *T. annulata *and *T. parva *[7], transcriptional data indicates that unlike *SfiI sub-telomeric *genes, *SVSP *family genes of *T. annulata *are expressed in a stage-specific manner by the macroschizont [7] and that the majority of the gene products contribute to the secretome [1,7] and see Additional file 2. While *SVSP *genes bear general similarity with *TashAT *genes, by both encoding a signal peptide and the previously defined FAINT domain, unlike TashATs they do not bear clear functional motifs shared across family members [7]. *SVSP *genes also show atypical codon usage compared to the *TashATs *and other major secretome gene families. A preliminary analysis was undertaken to investigate diversity by focussing on differential codon usage among secretome gene families. The set of macroschizont-expressed genes that encode secreted products was analysed using correspondence analysis to investigate whether particular genes showed bias in codon usage (Figure 3). The results revealed a cluster of genes with atypical codon usage on the right hand side of the graph and these were identified as *SVSP *genes. Relative Synonymous Codon Usage (RSCU) is the ratio of the observed frequency of a codon relative to that expected if codon usage is uniform, i.e. values tending towards one indicate an absence of bias. When correspondence analysis was performed using RSCU, the *SVSP *genes continued to cluster separately (data not shown) indicating that the atypical codon usage was independent of amino acid composition. In contrast to *SVSP*s, *TashAT *genes show no bias in codon usage. The finding of codon bias in *SVSP *genes could be explained by translational selection for the use of optimal codons as seen in trypanosome parasites [21], and may be a requirement for elevated synthesis or translational control of SVSP proteins.

On the basis of the results outlined above, the *SVSP *family was selected for allelic sequencing in order to compare the results with that of *TashAT *family. Thus, these gene families represent polypeptides of the *T. annulata *secretome, which are subject to positive selection at the inter-species level although available genomic evidence suggests they are likely to perform contrasting functions. This study was undertaken to test the hypothesis that differential selection pressures have operated during evolution of the *TashAT *and *SVSP *gene families and that this relates to the potential biological function that the proteins of these respective families perform.

### Allelic sequencing of selected members of the *T. annulata SVSP *and *TashAT *gene families

Four *SVSP *genes, denoted *SVSP1 (TA16025)*, *SVSP2 (TA17485)*, *SVSP3 (TA17545) *and *SVSP4 (TA16045) *were selected for sequencing to investigate allelic diversity within a panel of Tunisian and Turkish isolates of *T. annulata *(see Additional file 3). All four genes have direct orthologues in *T. parva*, with relatively high d_N_d_S _values ranging from 0.1796 for *SVSP3 *to 0.4037 for *SVSP1 *(see Additional file 4). *SVSP1 *and *SVSP4 *are located in the same sub-telomere in chromosome II of *T. annulata *(Figure 2) while *SVSP3 *(chromosome III) and *SVSP4 *(chromosome I) encode a predicted nuclear localisation signal. The latter genes were selected in order to investigate whether diversity for nuclear-targeted *SVSP *genes (n = 5) differed from the majority of *SVSP*s which lack a predicted NLS motif. Four members of the *TashAT *family were also selected for allelic sequencing, each of which is expressed in the macroschizont and encode signal peptides together with an NLS motif. TashAT2 (TA20095), TashAT3 (TA20082) and SuAT1 (TA03135) each bear a different number of AT-hook DNA binding motifs: one for SuAT1, three for TashAT2 and four for TashAT3 [7,13,14,22]. Additionally, *TashHN *(TA20090) allelic sequences were obtained because, together with *TashAT2*, *TashHN *flanks the gene cluster [1] and shows a high level of identity across predicted functional motifs (including NLS) with its *T. parva *orthologue [23]. Based on this result it was predicted that TashHN performs a conserved function and will show low divergence within a species. Tunisian and Turkish *T. annulata *DNA samples were used as templates for PCR amplification (see Additional file 3) and multiple allelic sequences were identified for each locus. Near full-length sequences were generated for all four *SVSP *genes, *TashHN *and *SuAT1*, while 20% and 32% coverage respectively was obtained for *TashAT2 *and *TashAT3 *sequences, corresponding to the AT-hook domain of these proteins.

### Analysis of the allelic sequences of four SVSP genes indicates they are highly diverse and are evolving neutrally

**Figure 4:**
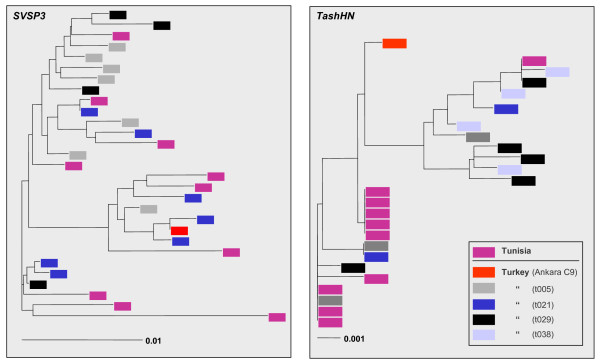
**DNA neighbour-joining trees**. Neighbour-joining trees were generated using allelic nucleotide sequence corresponding to eight loci (*SVSP1*, *SVSP2*, *SVSP3*, *SVSP4*, *SuAT1*, *TashHN*, *TashAT2 *and *TashAT3*). Representative trees for *SVSP*s (*SVSP3*) and *TashAT *family genes (*TashHN*) are illustrated with leaves colour-coded to differentiate alleles representing Tunisian clones and each of the four highly heterogeneous Turkish isolates.

A summary of the allelic sequencing results is shown in Table 2. Between 21 and 30 alleles were identified for each *SVSP *member and at each locus considerable length polymorphism was evident. Allelic amino acid sequences were aligned and each site containing a gap was removed from the alignment (indel sites are illustrated in Additional file 1). For *SVSP *genes, the number of polymorphic sites identified in the un-gapped alignments broadly correlated with the length of each gene, with the largest gene (*SVSP1*) possessing 151 polymorphic sites. Nucleotide diversity ranged between 1.4% for *SVSP3 *and 2.8% for *SVSP1*. To investigate whether allelic variation was related to geographical origin, neighbour-joining trees were constructed for each gene, which included identical alleles generated from different DNA templates and an example for *SVSP3 *is illustrated in Figure 4. Minimal geographical sub-structuring is evident, with alleles from Tunisian and Turkish samples interspersed on the tree. A similar pattern was shown for *SVSP2 *(data not shown) and this is reflected in low levels of molecular variance among populations for these two genes (Table 2). *SVSP1 *and *SVSP4 *showed limited evidence of geographical sub-structuring and higher levels of molecular variance among populations, however the alleles from each country did not cluster discretely (data not shown) and therefore overall there is no clear evidence for geographical sub-structuring of *SVSP *alleles.

**Table 2:** Summary of sequencing results, neutrality tests and AMOVA

Gene name	Amplicon length (bp)	Coverage of C9* by ungapped consensus	Molecular variance among populations	Overall diversity & neutrality							Diversity & neutrality in Turkey						
				n	S	π	θ_π_	*D* (Tajima)	*D* (Fu & Li)	*F* (Fu & Li)	n	S	π	θ_π_	*D* (Tajima)	*D* (Fu & Li)	*F* (Fu & Li)
*SVSP1*	551 - 576	89%	26%	23	151	0.028	41	-0.192	-0.947	-0.834	12	111	0.028	37	0.389	0.011	0.126
								*0.371*	*0.425*	*0.483*					*0.391*	*0.427*	*0.453*
*SVSP2*	409 - 414	97%	1%	21	101	0.025	32	-0.437	-0.520	-0.579	13	92	0.024	30	-0.370	-0.039	-0.147
								*0.435*	*0.508*	*0.561*					*0.415*	*0.458*	*0.495*
*SVSP3*	408 - 420	94%	3%	29	104	0.014	27	-1.548	-2.888	-2.889	19	53	0.011	15	-0.055	-0.858	-0.859
								*0.456*	*0.622*	*0.601*					*0.620*	*0.731*	*0.800*
*SVSP4*	476 - 508	92%	25%	30	138	0.021	35	-0.767	-1.749	-1.676	22	116	0.022	33	-0.405	-0.870	-0.851
								*0.422*	*0.509*	*0.557*					*0.439*	*0.500*	*0.536*
*TashHN*	333 - 337	100%	18%	16	20	0.006	6	0.155	-0.677	-0.510	10	13	0.005	5	0.137	-0.263	-0.184
								*0.942*	*0.972*	*1.045*					*1.066*	*0.912*	*1.122*
*SuAT_1_*	463 - 494	81%	71%	16	100	0.031	30	**1.621**	**1.025**	**1.381**	8	52	0.012	20	-1.031	-1.299	-1.379
								*0.440*	*0.484*	*0.567*					*0.928*	*0.898*	*1.015*

The number in italics below the value for each test is the upper limit of the 95% confidence interval, calculated by coalescence simulation based on θ_π _and free-recombination, n = number of sequences compared, S = number of segregating sites, π = nucleotide polymorphism and θ_π _= theta (an estimator of genetic variation) based on nucleotide polymorphism. * C9 is the name of the *T. annulata *Ankara clone used in the genome sequencing project. Results for *TashAT2 *and *TashAT3 *were not generated as the large number of gaps in the alignment precluded a meaningful analysis.

Several tests of neutrality were undertaken for each gene using both the overall allelic dataset and then using Turkish-derived alleles alone (Table 2). For each *SVSP *gene, negative test values were returned and only in the case of Turkish alleles of *SVSP1 *were low positive scores obtained, 0.389 for Tajima's *D *test and 0.011 and 0.126 for Fu and Li's *D *and *F *tests respectively. For each gene, none of these values exceeded the upper 95% confidence limit for a neutrally evolving population as determined by coalescence simulation. On this basis, there is no evidence for positive selection acting on these genes within the two populations analysed.

### TashAT alleles exhibit geographical sub-structuring

For the *TashAT *genes in *T. annulata*, 16 distinct alleles were identified for both *TashHN *and *SuAT1 *and similar to the *SVSP*s, length polymorphism was identified among all *TashAT *genes (Table 2). The AT-hook domain of *TashAT2 *(33 alleles) and *TashAT3 *(9 alleles) showed the greatest level of length polymorphism with proteins differing by up to 108 and 47 residues respectively. Among the TashHN alleles, a single four amino acid indel was identified which contrasts with the large number of insertions and deletions evident towards the 5' end of *SuAT1 *(see Additional file 1). Overall, *TashHN *showed only 20 polymorphic sites together with the lowest average number of nucleotide differences per site (6). *TashAT2 *and *TashAT3 *contained a complex pattern of indels that precluded the generation of a meaningful un-gapped alignment. In contrast to *SVSPs*, for both *SuAT1 *and *TashHN *geographical sub-structuring of the allelic sequences is evident, with the Turkish and Tunisian alleles tending to cluster independently and this is illustrated for *TashHN *in Figure 4. Unexpectedly, identical alleles were found among Tunisian samples, even though they were isolated from different geographical locations and a single outlying Tunisian allele was identical to a Turkish allele. *SuAT1 *showed the highest level of DNA polymorphism of all genes in the study (π = 3.1%) and the greatest geographical sub-structuring with 71% of the molecular variation in the allelic dataset directly attributed to differences between Tunisian and Turkish alleles. Contrasting results were obtained with the neutrality tests between *TashHN *and *SuAT1*. For *TashHN*, in the Turkish allelic dataset and the entire population, a low positive Tajima's *D *statistic together with negative values for Fu and Li's tests were calculated, although the low number of alleles and the minimal sequence diversity limited the power of these tests. For *SuAT1*, positive neutrality test values were calculated for the entire allelic dataset and a positive Tajima's *D *statistic indicated a low level of both low and high frequency polymorphisms. This may be interpreted as evidence of a decrease in population size or balancing selection, but can most readily be explained by the evident geographical structuring of *SuAT1 *alleles. This is supported by the fact that all three neutrality test statistics became negative when the Turkish population was analysed in isolation (Table 2).

### Although highly diverse, SVSP alleles appear not to be positively selected

**Figure 5:**
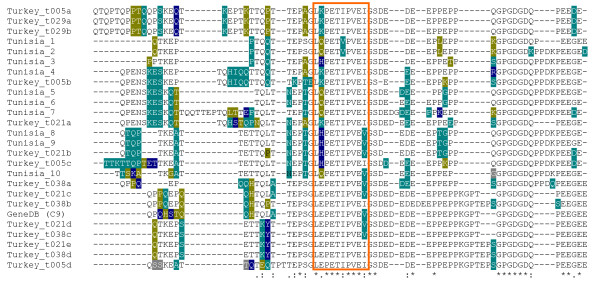
**Hypervariable region of SVSP1**. Amino acid sequences representing alleles of SVSP1 were aligned, identifying hypervariability in the PEST domain in the centre of the protein. At each site the second (green), third (gold), fourth (blue) and fifth (grey) most frequent amino acids are indicated and the conserved motif L(Q/*)PET(V/I)PVE(V/I) is highlighted.

Allelic d_N_d_S _values were calculated for each of the six genes in *T. annulata *(Table 3). All *SVSPs *displayed similar ratios with values ranging from 0.350 to 0.547, each gene displaying higher values compared to the inter-species comparison. All the *SVSP *genes showed a higher number of negatively selected sites than positively selected. A codon-by-codon analysis of each *SVSP *gene revealed that positively and negatively selected codons are distributed throughout the protein, with little evidence of clustering (data not shown). To test for evidence of positive selection within *T. annulata *compared with *T. parva*, the McDonald-Kreitman test was undertaken for each gene. This test measures whether synonymous or non-synonymous differences are skewed within or between species and this is expressed as a neutrality index showing an excess or deficit of within-species non-synonymous polymorphisms. Under neutrality, the ratio of non-synonymous to synonymous substitutions between species will be equal to the ratio obtained within a species. The results are shown in Table 3, and for the *SVSP *genes the obtained values ranged from 0.754 for *SVSP4 *to 0.834 for *SVSP2 *suggesting that these genes are evolving neutrally. Multiple indels were identified within SVSP alleles particularly in PEST domains and an amino acid alignment of the region of *SVSP1 *hyper-variable for indels and amino acid substitutions is shown in Figure 5. A complex pattern of insertions and deletions was identified in this region that is rich in proline (P), glutamine (Q), aspartic acid (D) and glutamic acid (E). Indels were found in PEST domains of each of the four *SVSP *genes. In direct contrast, a highly-conserved motif, LEPETIPVEIGSDED, was identified in the midst of this polymorphic region and its relative position varied within the proteins as a result of the indels (Figure 5). The codons encoding the first, third and ninth residues in this motif show synonymous substitutions and together with the monomorphic codons, the data suggest that the motif may be under purifying selection. Interestingly, LEPETIPVEIGSDED related motifs are found in a total of 16 SVSP predicted proteins encoded by different paralogous genes. The motif also shows strong conservation within the TashAT gene family (14 of 17 paralogous sequences), including SuAT1 and TashAT2 and TashAT3, and can be present in up to 4 copies located in different regions of the predicted polypeptides [13].

**Table 3:** d_N_d_S _and McDonald-Kreitman results

Gene name	No. of sequences analysed	Positively selected sites (*p < 0 .25*)	Negatively selected sites (*p < 0.25*)	*T.a. *allelic d_N_d_S_	*T.a. *vs *T.p. *d_N_d_S_	Polymorphic changes within *T. annulata*		Fixed differences between species		Neutrality index	*p value *(Fisher's exact test)
						Syn	Nsyn	Syn	Nsyn		
*SVSP1*	23	8	27	0.547	0.404	42	108	113	361	0.805	0.329
*SVSP2*	21	5	23	0.464	0.312	36	69	127	292	0.834	0.480
*SVSP3*	29	4	21	0.369	0.180	37	63	133	288	0.786	0.343
*SVSP4*	30	2	12	0.350	0.209	45	77	148	336	0.754	0.186
*TashHN*	16	0	5	0.211	0.265	11	9	84	139	0.494	0.153
*SuAT_1_*	16 (total)	0	9	0.502	0.219	16	49	174	353	1.510	0.205
*SuAT_1_*	8 (Tunisian)	0	3	0.611	0.219	8	27	178	359	1.673	0.265
*SuAT_1_*	8 (Turkish)	0	4	0.478	0.219	9	27	175	358	1.466	0.364

*T. annulata *sequences compared with *T. parva*, Syn = number of synonymous changes, Nsyn = number of non-synonymous changes

### TashAT genes show evidence of purifying selection and conservation of functional motifs

**Figure 6:**
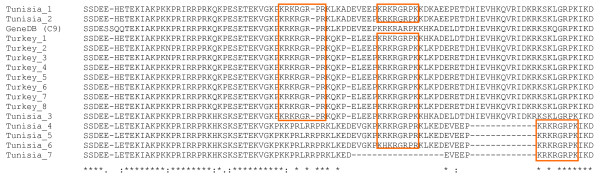
**DNA binding motifs in SuAT1**. Amino acid sequences representing alleles of SuAT1 were aligned and DNA binding motifs termed 'AT-hooks' are highlighted in orange. Block differences distinguish the five Tunisian alleles at the bottom of the alignment and the Turkish-derived alleles. The Tunisian sequences Tunisia_1 and Tunisia_2 and the C9 sequence (Turkey) at the top of the alignment are intermediate between the archetypal Tunisian and Turkish alleles.

Contrasting results were obtained for *TashHN *and *SuAT1 *in *T. annulata *using tests for positive selection. *TashHN *was found to be highly conserved at the allelic level and was the only gene where more synonymous than non-synonymous changes were identified (11 vs 9, Table 3) with a low total number of mutations reflecting limited nucleotide diversity (0.6%, Table 2). When compared with *T. parva*, an excess of non-synonymous substitutions was identified relative to synonymous substitutions at an inter-species level and this resulted in a lower neutrality index than that of *SVSP*s. *SuAT1 *showed elevated d_N_d_S _at the intra-species level, although no statistically significant positively selected codons were identified. An excess of non-synonymous substitutions were detected within *T. annulata *compared to synonymous substitutions (49 vs 16) and when compared to the fixed differences between species gave an elevated neutrality index of 1.510 (Table 3). To test whether geographic sub-structuring of allelic types could account for this result, the analysis was repeated, with each country tested separately, and this revealed similar d_N_d_S _and neutrality indices to those from the whole dataset (Table 3). This shows it is likely that within-population selection has occurred, although the value for the neutrality index was not statistically different from 1. Analysis of an alignment of *SuAT1 *alleles over the AT-hook region of the protein showed remarkable conservation of these motifs but revealed differences between the predicted proteins derived from Tunisia and Turkey. AT-hooks are small motifs with a related sequence pattern rich in arginine (R) and lysine (K) around a central GRP core that binds to the minor groove of short stretches of AT-rich DNA [24]. The majority of SuAT1 alleles (14/16) show a double AT-hook arrangement with the first AT-hook showing a RGRPR motif while the second AT-hook has a RGRPK motif (Figure 6). The spacing between the motifs is strongly conserved with the majority of Turkish alleles displaying a spacing of 14 amino acids between the GRP core, while Tunisian alleles show spacing of 15 residues. In the alignment shown in Figure 6, where amino acid substitutions disrupt the first AT-hook in four of the Tunisian alleles (Tunisia_4 to Tunisia_7), with a single exception, additional substitutions compensate by reconstituting the basic double AT-hook pattern displayed by the majority of alleles. Two sequences showed variants of this basic pattern: the C9 (genome strain) allele encodes a protein that posses only AT-hook 1 and a Tunisian allele encodes a protein that only posses AT-hook 2 (Tunisia_7). An additional upstream NLS is also highly conserved, with only a single di-morphic amino acid residue identified among alleles (data not shown). The LVPETIPVEIGSDEE motif, starting at position 352 is completely conserved across SuAT1 alleles with a single synonymous mutation evident encoding the valine residue in position eight (data not shown). It may be concluded from the two fully-analysed *TashAT *family genes, that despite a degree of allelic polymorphism, there is evidence of major conservation over the entire encoded protein (TashHN) and specifically there is conservation of known functional motifs (SuAT1).

**Figure 7:**
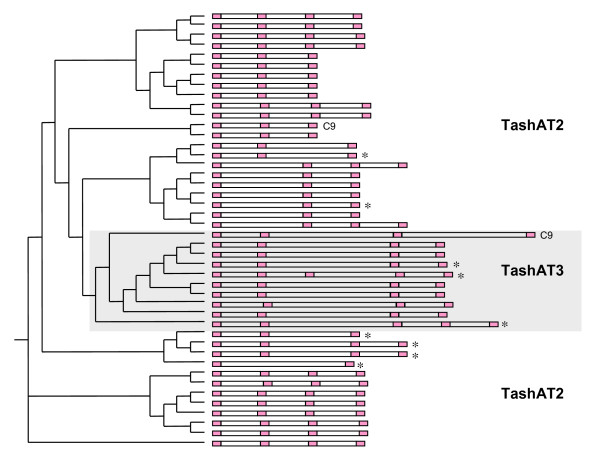
**TashAT2 and TashAT3 AT-hook domains**. The AT-hook domains of TashAT2 and TashAT3 were aligned and used to generate a neighbour-joining tree. The figure illustrates the number of AT-hooks (indicated in pink) and the distance between core 'GRP' motifs for each allele. Tunisian sequences are indicated by an asterisk.

The high number of indels in the *TashAT2 *and *TashAT3 *sequences precluded formal d_N_d_S _analysis. Despite this variation, AT-hook motifs were found to be highly conserved across all allelic sequences. To investigate the relationship of these domains in TashAT2 and TashAT3, a neighbour-joining tree was created based on the entire AT-hook region of allelic sequences (Figure 7). *TashAT3 *alleles cluster together, the majority encoding predicted polypeptides with four AT-hooks and two Tunisian alleles encoding five. Similarly, the majority of *TashAT2 *alleles encode either three or four AT-hooks, with a single Tunisian allele encoding only two. As with SuAT1, the spacing between AT-hooks remained constant, with the majority of core motifs separated by 14 residues. Over the entire dataset, spacing between AT-hook motifs was distributed around three basic sizes: 14 (± 1), 29 (+1) and 44 residues. The relative positioning of pairs of hooks and associated spacing showed variation across the predicted proteins, with evidence of a mosaic type pattern. Thus, the possession of two or more AT-hook motifs in these sequences shows absolute conservation but the patterning of AT-hooks for different TashAT polypeptides shows considerable variability. A degree of geographical sub-structure is evident in *TashAT2 *alleles, with four of the six Tunisian sequences clustering (Figure 7).

## Discussion

Large multi-gene families located at the sub-telomeres of protozoan parasite genomes often encode antigens that display sequence hyper-variability to allow escape from a protective immune response, while genes encoding proteins that engender adaptation to particular host environments are more likely to be conserved within-species. In this study we have analysed allelic diversity of members of two distinct families of proteins of *T. annulata *that are predicted to be secreted into the host compartment and have been considered as candidate parasite molecules that enable evasion of a protective immune response (*SVSP*s [20]) or function to control host cell phenotype (*TashAT*s [14,22]). *SVSP *genes are arranged in sub-telomeric arrays while all the *TashAT*s are located in an internal cluster on chromosome one.

Allelic sequencing identified a high level of diversity at the nucleotide and amino acid level across the length of all four *SVSP *genes analysed, with hyper-variability identified at the site of the predicted PEST degradation motifs (see Figure 5). However, the combined analyses performed in this study indicate that the *SVSP *genes are evolving in a neutral manner. The McDonald-Kreitman test, specifically designed for detecting intra-specific selection, also indicated these genes have not been subject to a strong positive selective pressure (see Table 2) with Tajima's *D *and Fu and Li's *D *and *F *tests failing to provide any evidence of a deviation from neutrality. Thus, with a large proportion of neutral mutations, SVSP genes appear to be evolving in the absence of strong purifying selection within *T. annulata *populations. In contrast to the findings for *SVSP*s, the level of allelic diversity of the four TashAT family genes that were analysed was, in general, less extensive with evidence for strong conservation of the predicted AT-hook functional motifs. Moreover, analysis of the *TashHN *sequences provided significant data for purifying selection to conserve amino acid sequence at the intra-specific level, while the *SuAT1 *alleles showed evidence for positive selection, with a greater number of non-synonymous mutations compared to synonymous mutations resulting in a higher within-species ratio and a neutrality index greater than one. We conclude that it is likely that the differences in the pattern of allelic diversity in extant populations displayed by the representative loci of these two major secretome gene families are a consequence of different evolutionary pressures that reflect contrasting functional properties of the proteins they encode.

Motif prediction analysis suggests the majority of SVSP proteins are secreted into the leukocyte cytoplasm [7] and it can be postulated that they play a role in the interaction between the parasite and the infected host cell. In addition, many *SVSP*s in both *T. annulata *and *T. parva *encode predicted nuclear localisation NLS motifs, and the vast majority possess the 'frequently associated in *Theileria*' FAINT domain, characteristic of proteins that constitute the macroschizont secretome. The conservation of the NLS in the allelic sequences of SVSP 3 and 4 identified in this study indicate this motif is functionally conserved, supporting the recent demonstration in *T. parva *that a typical SVSP protein can locate to the nucleus of transfected mammalian cells [20]. In addition, the allelic sequence data highlighted the L-PETIPVEIGSDED motif notable for its level of conservation in the midst of a region that was shown to be divergent. Related motifs can be identified in a number of predicted proteins of the *T. parva *secretome (data not shown) and nearly all TashAT family members [1]. Despite the identification of conserved motifs, a biological function for SVSPs has yet to be proposed. Although a *T. parva*-encoded SVSP (TP03_0882) was shown to locate to the nucleolus in transfected U2OS cells, a host nuclear/nucleolar location for SVSPs in *Theileria *infected cells was not demonstrated [20]. Indeed, detection of macroschizont reactivity by an anti-SVSP serum was limited to a small number of cells and endogenous polypeptide was not detected by immunoblotting [20]. This is in stark contrast to members of the TashAT cluster analysed in this study, which have been shown to be present at significant levels in the nucleus of the majority of *T. annulata *macroschizont-infected leukocytes [13,14].

One explanation for the difficulty in detecting SVSPs is that these proteins are likely to be rapidly degraded within the host compartment, and the possession of multiple PEST motifs that are known to target eukaryotic proteins for proteolytic degradation supports this. The presence of such motifs and signal peptide on SVSPs is compatible with the hypothesis that these proteins are secreted into the host compartment, degraded and subsequently presented as peptides on MHC Class I molecules [25]. Recognition of class I presented peptides by cytotoxic T cells (CTL) has been shown to play an important role in protective immunity against *T. parva *[26] and an SVSP family member has been identified among a panel of T cell antigens in this species (I. Morrison, personal communication). The recognition of pathogen peptides by CTL is known to exert an immune selection pressure that results in selection of amino acid substitutions in critical residues of the epitope [27], and evidence for diversifying selection of a predominant (non-SVSP) T cell epitope of *T. annulata *has been obtained (Weir and Morrison, unpublished data). However, despite showing a significant level of allelic diversity, none of the *T. annulata SVSP *gene sequences analysed in this study provide evidence to support the hypothesis that divergent allelic forms of the SVSP proteins studied have evolved to escape recognition by CTL. Nevertheless, it has been argued that *Theileria *CTL antigens may be subject to weak selection as a result of bovine MHC Class I polymorphism [28] and therefore this possibility cannot be completely discounted.

The *SVSP *family of genes are located at the sub-telomeric regions of the chromosomes and show evidence of expansion and diversification in *T. annulata *and *T. parva*. A telomeric location of gene families can promote ectopic recombination and the presence of a large number of indels across *T. annulata SVSP *allelic sequences (see Figure 5) suggests that generation of sequence diversity via recombination could be a relatively frequent event, at least in the family members studied. These characteristics bear similarity to families of sub-telomerically located genes in other protozoan parasites that perform an essential biological function but, due to exposure to a protective immune response, have evolved into multiple divergent antigenic forms. Variant expression of different antigenic types allows escape from protective immunity, and the genes act as a contingency against parasite killing. The two most extensively studied systems are the *vsg *[29] and *var *[30] gene families of Trypanosome and *Plasmodium *parasites, respectively. The manner in which these contingency gene families have evolved has been difficult to assess and consequently there is no experimental data for or against diversifying selection or neutrality. Moreover, since it is possible that selection against contingency genes operates, in principle, against the whole antigenic repertoire, the finding of neutrality for allelic diversity of *SVSP*s does not exclude a role for *SVSP*s as a contingency gene family. Indeed, the observation that *SVSP *sub-telomeric gene location bears similarity to *vsg *and *var *gene families has been made and it was proposed that restricted expression of individual *SVSP*s could be responsible for the small number of parasites expressing detectable SVSP antigens [20].

For *SVSP*s to act as a family of classical contingency genes, like the *vsg *and *var *genes, it can be expected that *SVSP *mRNA expression patterns would show evidence of variant expression between distinct parasite genotypes and over time. However, transcription profiling of *SVSP *genes in both *T. annulata *and *T. parva *infected cell lines does not fit the classical pattern of variant expression, as the majority of *SVSP *genes are co-expressed at the RNA level [7,20]. In addition, comparison of different infected cell lines showed that the *SVSP *expression profile was largely comparable and did not vary over time. While the possibility of differential translational control or protein stability cannot be discounted, an alternative model that merits consideration is that in all macroschizont infected cells, the majority of SVSP proteins are continually generated and rapidly degraded. If such a situation occurred then each infected cell would simultaneously generate a plethora of random variant peptides with the potential to be presented at the host cell surface with either class I or class II MHC antigens, assuming the SVSPs contain the relevant motifs for presentation. The consequence of this large peptide repertoire on subversion of the host immune response to these proteins is unknown, but it would be reasonable to predict that a wide range of peptide specificities would be generated with each peptide present at a relatively low frequency. Like the contingency theory, selection for a particular amino acid variant may not operate, as it is the generation of the divergent pool itself that confers a selectable advantage. Investigation of *SVSP *gene expression at the level of the single infected cell and evidence for stimulation of cells of the immune response by SVSP peptides will be required to provide supporting evidence for the postulation that the *SVSP *family has evolved to subvert the bovine immune response.

In direct contrast to the results obtained from analysis of *T. annulata SVSP *genes, *TashHN *was found to have very limited allelic diversity within the species. Indeed, several codons were found to be under the influence of negative selection whereas none showed evidence of positive selection. The lack of diversity within *T. annulata *is not unexpected as there is a high level of sequence identity with the *T. parva *orthologue and predicted functional motifs are located in identical positions within the polypeptide [23]. Based on this, it is proposed that an ancestral gene with significant identity to *TashHN *was present in the common ancestor of *T. annulata *and *T. parva*. TashHN has no identified DNA binding domains but is known to locate to the leukocyte nucleus [31]. Evidence for phosphorylation of the protein has been generated and its expression levels are significantly elevated in infected cell lines that show an attenuated phenotype [31]. In conclusion, the results obtained in this study strongly support the hypothesis that the function of the TashHN protein as a modulator of host cell phenotype is conserved across transforming *Theileria *species and predicts that sequence diversity would also be limited in *T. parva*.

Unlike *TashHN*, *T. annulata TashAT *family genes encoding polypeptides possessing AT-hook motifs (*TashAT1-3 *and *SuAT1-3*) show significant divergence from their *T. parva *orthologues, which completely lack AT-hooks. This finding could be interpreted as indication that the AT-hook motifs do not perform a critical biological function. However, the results of this study clearly imply that species-specific functional divergence of *TashAT *family genes has occurred following the *T. annulata*/*T. parva *split, as strong conservation of the AT-hook motifs was observed in all TashAT and SuAT1 sequences analysed in this study. Further studies are required to demonstrate whether motifs specific to the *T. parva *orthologues are conserved across alleles. Interestingly, the TashAT3 allele cluster does not segregate at the first branch of the tree displayed by Figure 7, suggesting that the ancestral TashAT3 may have been more closely related to particular alleles of TashAT2, and that complete sequence divergence has not occurred at these loci. For the majority of SuAT1 alleles, strong conservation of the sequence defining AT-hook 1 and 2 was found, together with conservation of the spacing between the motifs. This suggests that binding to the target AT-rich sequence is an essential function of this protein. In mammalian systems, specificity of promoter recognition by AT-hook proteins has been attributed to differences in core motif sequence and spacing between motifs: the HMGA1 protein, for example, has three AT-hooks and specifically binds to tandem repeats of AT-rich sequence in the INF-β gene using the central AT-hook in combination with either the first or third hook [32]. The arrangement of AT-hook motifs in TashAT sequences (Figures 6 and 7) shows similarity to HMGA proteins and the AT-hook region of TashAT2 has been shown to bind tandem repeats of AT-rich DNA [14]. We conclude that the results of this study support the postulation that members of the TashAT gene family have evolved to modulate the activation outcome of the host cell lineage preferentially infected by *T. annulata*.

Despite displaying strong conservation of functional motifs, variation was identified across TashAT/SuAT allelic sequences. Firstly, TashAT2 and 3 alleles display extensive variability over the AT-hook region with allelic variants having between one and five hook motifs. There is also variation in the distance between pairs of hook motifs, fixed around three gap sizes, and different alleles show different combination of AT-hook gaps. Variation in number of and spacing between AT-hook motifs has been documented for a number of proteins in different organisms and is thought to be associated with distinct target recognition [32]. AT-hooks have been described as evolutionary mobile modules dispersed by translocation of complete and contiguous units of the motif [24] and our results support this model. Why *T. annulata *exhibits this level of diversity in DNA binding proteins implicated in modulation of host cell gene expression is not clear. In this context, further allelic sequence analysis of other members of the gene family will determine whether these conclusions apply to the family as a whole. It is of interest to note that different *T. annulata *and *T. parva *infected cell lines show significant variability in the profile of cytokine genes they express [33,34], despite activation of the same host cell transcription factors [35].

Geographical sub-structuring of *TashHN *and *SuAT1 *allelic sequences (Figures 4 and 6) was identified in this study. The simplest explanation for this divergence is isolation and genetic drift, whereby mutations in the gene arising in separate areas eventually resulted in different allelic types. However, to date clear sub-structuring of divergent allelic sequences appears to be restricted to *TashAT *family genes, as this property is limited or absent for all other loci analysed to date. In addition, the evidence of positive selection of SuAT1 sequences within the species is most likely to be due to the evolution of two distinct groups of SuAT1 alleles that show significant differences in amino acid sequence, but maintain the predicted basic functional motifs. A more radical hypothesis is that evolution of TashAT family proteins has been driven by recognition of differences in chromatin targets and that these have evolved to be subtly different in cattle or buffalo indigenous to geographically distinct regions endemic for *T. annulata*.

## Conclusions

The results of this study indicate that contrasting selection pressures have shaped the evolution of two gene families encoding secretome proteins of *T. annulata *and that these differences are likely to relate to the function of the encoded proteins. *SVSP *genes have evolved to be divergent (or perform a function that has limited requirement for amino acid conservation) and a logical prediction is that SVSP proteins interact at an undefined level with the bovine immune system. In contrast, TashHN shows a high level of constraint and may function to alter a property of the host leukocyte that is fundamental to infection by both *T. annulata *and *T. parva*. Finally, *TashAT *genes are constrained within, but not between, species and show clear evidence of divergence in the arrangement of conserved functional motifs. These proteins are most likely to perform a species-specific function that allows adaptation to infection and activation of the preferred host cell for each species and, more controversially, host genetic backgrounds.

## Methods

### Genomic analysis

The published genomes of *T. annulata *and *T. parva *were used as a primary resource for this study [7,23]. Orthologous relationships between genes were defined in the initial comparative genomic analysis [7] using a reciprocal BLASTing method and this information is available via the *T. annulata *genome browser [19]. To confirm these results within the gene families under study, a combination of methods was used including phylogenetic analysis. ClustalX [36] was used for aligning both nucleotide and amino acid sequences and PHYLIP trees generated from alignments were viewed using TreeViewX [37]. Additionally, chromosomal sequences were directly compared using the Artemis Comparison Tool [38] in order to investigate synteny. Expressed sequence tag (EST) data were available for the macroschizont, merozoite and piroplasm stages of *T. annulata *(10,000 reads per stage), which mapped to a total of 2,078 genes across the 3,793 coding sequences in the genome [7]. The SignalP2.0 HMM algorithm was utilised to identify proteins which enter the secretory pathway on the basis of encoding a signal peptide [39] and an improved version, SignalP3.0 [40], was used in the analysis of *SVSP *genes. Trans-membrane protein topology was determined using a hidden Markov model algorithm [41] and GPI-anchored proteins were identified using proprietary software, DGPI v. 2.04 [42]. Proteins containing a nuclear localisation signal (NLS) were identified using PredictNLS software, which compares a query protein sequence with a set of known NLS, also identifying DNA binding motifs [43]. PEST motifs are defined as hydrophilic stretches of at least twelve amino acids with a high local concentration of the amino acids proline (P), glutamic acid (E), serine (S) and threonine (T), the presence of which considerably reduces the half-life of a protein [18,44]. These were identified using the PESTfind algorithm http://emboss.bioinformatics.nl/cgi-bin/emboss/pestfind. The Tribe-MCL protein clustering algorithm [45] was used to group *T. annulata *proteins into putative families. A list of the top 30 *T. annulata *family clusters can be found in Table S3 of the online supplementary data that accompanied publication of the genome [7]. Codon usage was analysed using the CodonW package http://codonw.sourceforge.net/. This software calculates several standard indices of codon usage and gene composition and can be used to identify putatively optimal codons and it also implements correspondence analysis. Correspondence analysis is a data ordination technique, which can determine the major trends in the variation of the data and may be used to distribute genes along continuous axes in accordance with identified trends. Such an approach is necessary to summarise and explain the complex variation that may be encountered when analysing codon usage among a large number of genes.

### Allelic sequencing

The origin and type of *T. annulata*-infected material are listed in Additional file 3. DNA was prepared from 300 μl of EDTA blood samples taken from four infected cattle from Turkey using the Wizard^® ^Genomic DNA purification system (Promega). Ten Tunisian macroschizont-infected cell lines were cultured in 25 cm^2 ^tissue culture flasks using Roswell Park Memorial Institute (RPMI) 1640 medium, supplemented with 15% foetal calf serum. Approximately 10^7 ^infected cells were centrifuged at 1500 g for five minutes and the cell pellet washed in phosphate buffered saline (PBS), re-suspended in PBS and DNA purified using a Qiagen QIAamp DNA Mini Kit. Previous multi-locus genotyping demonstrated that the Turkish field isolates represented multiple parasite genotypes, while the Tunisian cell lines each represented a single haploid genotype [46,47].

Forward and reverse PCR primers for each of six genes (*SVSP1*, *SVSP2*, *SVSP3*, *SVSP4*, *TashHN *and *SuAT1) *were designed in the signal peptide and 3' downstream sequences respectively, with only two exceptions: (i) the forward primer for *SVSP1 *was located just upstream of the translation initiation codon and (ii) an intragenic reverse primer was designed for *SuAT1 *for samples that would not amplify with the primer located in the 3' sequence. Primer design was based on the published genome [7] and the oligonucleotide sequences are listed in Additional file 5. PCR primers flanking the sequence corresponding to the AT-hook regions of *TashAT2 *and *TashAT3 *loci were also designed (see Additional file 5).

An aliquot of each DNA preparation was PCR amplified in a total reaction volume of 20 μl under conditions previously described [48], using a Techne TC-512 thermocycler with the following settings: 94°C for 2 minutes, 30 cycles of 94°C for 50 seconds, 50°C for 50 seconds and 65°C for 90 seconds, with a final extension period of 15 minutes at 65°C. A mixture of *Taq *polymerase and a proofreading polymerase (*Pfu*) at a ratio of 15:1 was used to improve the fidelity of the reaction and the PCR products were subsequently cloned into the sequencing vector pCR4^®^-TOPO^® ^(Invitrogen). For each gene, a number of colonies were selected and up to 20 μg of plasmid DNA was isolated from each culture using a proprietary kit (Qiagen). Turkish blood preparations were known to contain multiple genotypes and therefore eight colonies were selected for sequencing each gene. For each Tunisian isolate, a single colony was selected as each cell line was known to represent a single genotype. 2 μg of air dried DNA was prepared for use in each sequencing reaction, which was performed by MWG Biotech, Germany. M13 universal and reverse primer sites in the vector flanking sequence were used to generate sequence reads to provide at least 2× coverage of every nucleotide. A number of allelic sequences were determined for *SVSP1 *[Genbank: GU373065-GU373088], *SVSP2 *[Genbank: GU373089-GU373111], *SVSP3 *[Genbank: GU373112-GU373154], *SVSP4 *[Genbank: GU373155-GU373193], *TashHN *[Genbank: GU373194-GU373219], *SuAT1 *[Genbank: GU373220-GU373241], *TashAT2 *[Genbank: GU373242-GU373274] and *TashAT3 *[Genbank: GU373056-GU373064].

DNA sequence polymorphism was evaluated using DnaSP [49]. Fu and Li's *D *and *F *tests [50] and Tajima's *D *test [51] were performed, as described, and the confidence intervals of these neutrality test statistics were estimated by coalescence modelling. DNA sequence variation was measured within and between populations using the McDonald-Kreitman test [52]. To test whether the level of synonymous or non-synonymous polymorphisms deviated from the neutral prediction of equal numbers, within *T. annulata *or between species, Fisher's exact test of significance was applied to the results for each gene; a low *p *value reflecting a departure from neutrality. The 'neutrality index' odds ratio was also calculated for each locus [53] to indicate if there was an excess (ratio > 1) or deficiency (ratio < 1) of non-synonymous substitutions within alleles from the same species. This was used as a qualitative and quantitative indicator of the direction and degree of selection. A maximum likelihood method was used to detect amino acid sites under positive selection and to determine d_N_d_S _values across alleles [54] and this was performed using the HyPhy platform [55]. The d_N_d_S _ratio was determined for each codon and where d_N _was greater or less than d_S_, a *p *value was derived from a two-tailed binomial distribution to assess the significance. Analysis of molecular variance (AMOVA) was performed using 'Genalex6' [56] in order to investigate the distribution of genetic variation among allelic sequences and to determine the level of population differentiation. Pair-wise estimates of genetic distance among populations within each species were calculated using Φ_PT_, the proportion of variance among populations relative to total variance.

## Authors' contributions

WW participated in the design of the study, carried out the bulk of the molecular work, performed the analyses and drafted the manuscript. TK participated in sample collected and purification of genomic DNA. MB carried out molecular work, amplifying and cloning alleles of two loci. AT participated in the design and co-ordination of the study. BRS participated in the design of the study and helped draft the manuscript. All authors read and approved the final manuscript

## Supplementary Material

Additional file 1 **SVSP and TashAT genes selected for allelic sequencing**. A schematic representation of the eight genes chosen for allelic sequencing Click here for file

Additional file 2 **The SVSP family in *T. annulata***. Details of each SVSP family member in the *T. annulata *genome including EST expression data, bioinformatic motif predictions and information about orthologous genes in *T. parva* Click here for file

Additional file 3 **Parasite material**. The type and origin of parasite material used to prepare template DNA Click here for file

Additional file 4 **Genes selected for allelic sequencing**. Details of each of the genes chosen for allelic sequencing including orthologous genes in *T. parva*, interspecies d_N_d_S _values, EST expression data in *T. annulata *and bioinformatic motif predictions Click here for file

Additional file 5 **PCR and sequencing primers**. Oligonucleotide sequences for the PCR and sequencing primers used in this study Click here for file
