# Supplementary material for: Evolution and diversity of secretome genes in the apicomplexan parasite Theileria annulata
Source: BMC Genomics. 2010 Jan 18;11:42. doi: 10.1186/1471-2164-11-42 (PMC2826314; doi:10.1186/1471-2164-11-42)
Supplement: Additional file 1 — SVSP and TashAT genes selected for allelic sequencing. A schematic representation of the eight genes chosen for allelic sequencing [file 1471-2164-11-42-S1.PDF]

Additional file 1 - SVSP and TashAT genes selected for allelic sequencing

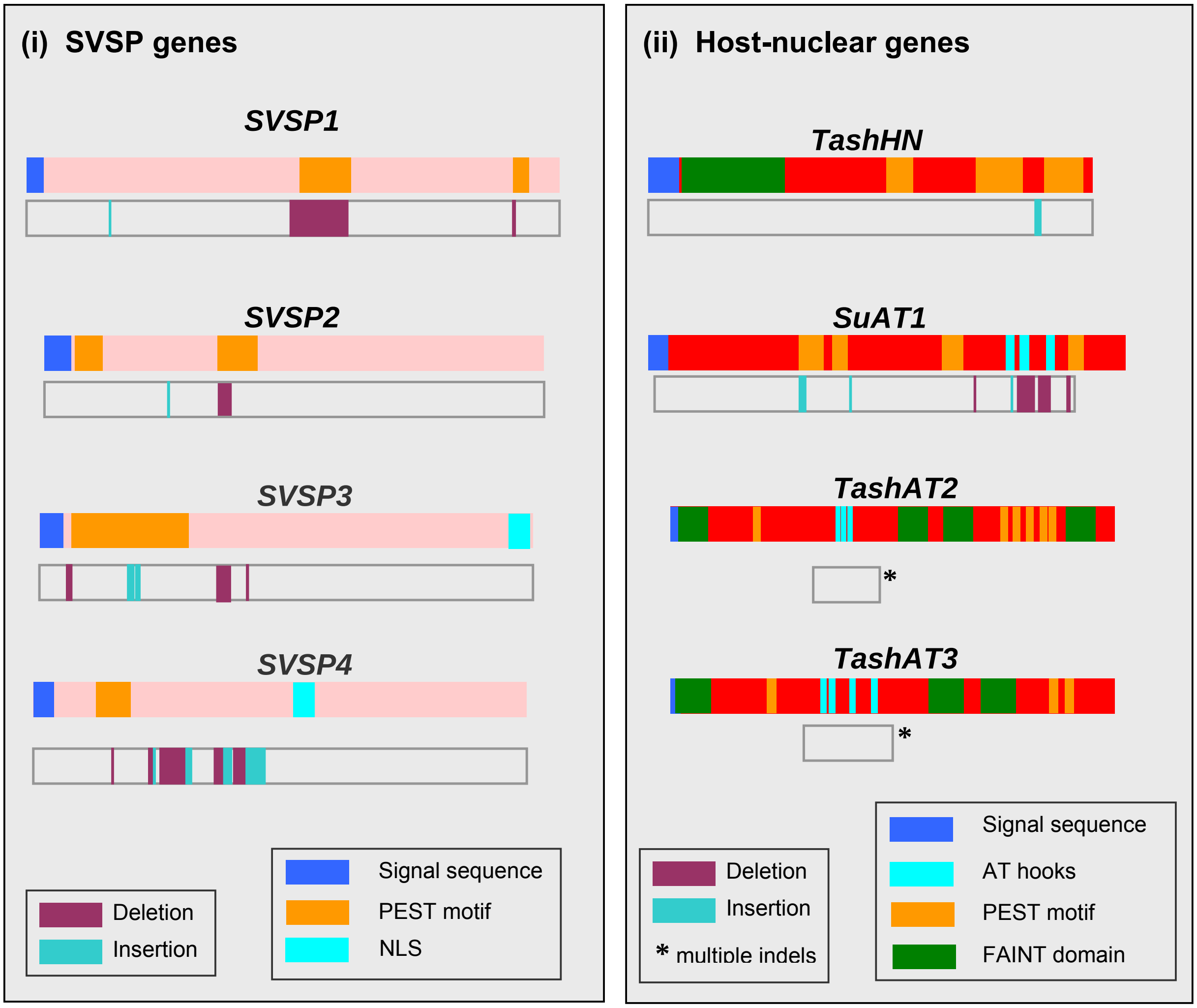

Eight genes were chosen for allelic sequencing, each of which exhibited length polymorphism. Only the AT-hook section of TashAT2 and TashAT3 were sequenced and multiple insertions and deletions were identified among the alleles with respect to the C9 genome.
