# Supplementary material for: Evolution and diversity of secretome genes in the apicomplexan parasite Theileria annulata
Source: BMC Genomics. 2010 Jan 18;11:42. doi: 10.1186/1471-2164-11-42 (PMC2826314; doi:10.1186/1471-2164-11-42)
Supplement: Additional file 2 — The SVSP family in T. annulata. Details of each SVSP family member in the T. annulata genome including EST expression data, bioinformatic motif predictions and information about orthologous genes in T. parva [file 1471-2164-11-42-S2.PDF]

Additional file 2 - The SVSP family in *T. annulata*

T. annulata ID	Locus	*T. parva* ID	$d_N d_S$	Protein identity (%)	Nucleotide identity (%)	Macroschizont EST data	Merozoite EST data	Piroplasm EST data	SignalP3.0	TMD	GPI	NLS	FAINT	PEST
TA18860	Ia	TP01_1227	0.0083	49.45	59.08	-	-	-	√	-	-	-	√	√
TA18865	Ia	TP01_1226	0.1584	48.83	63.94	-	√	-	√	-	-	-	√	√
TA18885	Ia	TP03_0890	0.1566	50.47	63.11	√	-	-	√	-	-	-	√	√
TA18890	Ia					√	-	-	√	-	-	-	√	√
TA18895	Ia					√	-	-	-	-	-	-	√	√
TA18950	Ia	TP01_0006	0.1846	47.89	65.05	√	-	-	√	√	-	√	√	√
TA19005	Ia					√	-	-	√	-	-	-	√	√
TA19060	Ia					√	-	-	√	-	-	-	√	√
TA17475	Ib					ND	ND	ND	√	-	-	-	ND	√
TA17480	Ib					-	-	-	√	√	-	-	√	-
**TA17485 _2_**	Ib	**TP01_1225**	**0.3118**	**44.97**	**62.4**	√	√	**-**	√	**-**	**-**	**-**	√	√
TA09420	IIa					√	-	-	√	-	-	√	√	√
TA09425	IIa	TP03_0886	0.0831	40.97	59.47	√	-	-	√	√	-	-	√	√
TA09430	IIa	TP02_0010	0.1572	45.45	61.93	√	-	-	√	-	-	-	√	√
TA09435	IIa	TP01_0005	0.1957	50.32	68.71	√	-	-	√	-	-	-	√	-
TA11385	IIa					√	-	-	√	-	-	-	√	√
TA11390	IIa					√	-	-	√	√	-	-	√	√
TA11395	IIa	TP02_0011	0.1137	43.72	63.71	-	-	-	√	-	-	-	√	√
TA11410	IIa					√	-	-	√	√	-	-	√	√
**TA16025 _1_**	**IIb**	**TP02_0955**	**0.4037**	**43.21**	**65.6**	√	**-**	**-**	√	**-**	**-**	**-**	√	√
TA16030	IIb	TP02_0956	0.1196	45.67	61.9	√	-	-	√	-	-	-	√	-
TA16035	IIb					-	-	-	√	-	-	-	√	√
TA16040	IIb	TP02_0958	0.2051	50.53	63.97	-	-	-	√	-	-	-	√	√
**TA16045 _4_**	IIb	**TP03_0001**	**0.2091**	**44.25**	**60.23**	√	**-**	**-**	√	**-**	**-**	√	√	√
TA02740	IIIa					√	-	-	√	-	-	-	√	√
TA17535	IIIa					-	-	-	√	-	-	-	√	√
TA17540	IIIa	TP02_0005	0.1378	46.05	59.49	√	-	-	√	-	-	-	√	-
**TA17545 _3_**	IIIa	**TP04_0002**	**0.1796**	**45.86**	**61.07**	√	**-**	**-**	√	**-**	**-**	√	√	√
TA17550	IIIa					√	-	-	√	-	-	√	√	√
TA17555	IIIa					-	√	-	√	√	-	-	√	√
TA05540	IIIb					√	-	-	√	-	-	-	√	√
TA05545	IIIb	TP03_0889	0.1501	43.62	61.26	√	-	-	√	-	-	-	√	√
TA05550	IIIb					-	-	-	√	-	-	-	√	√
TA05555	IIIb	TP03_0887	0.1594	36.6	58.93	√	-	-	√	-	-	-	√	√
TA05560	IIIb					√	-	-	√	-	-	-	√	√
TA05565	IIIb					√	-	-	√	-	-	-	√	√
TA05570	IIIb	TP03_0868	0.1967	46.59	64.47	√	-	-	√	-	-	-	√	-
TA05575	IIIb	TP03_0867	0.2097	47.59	67.69	√	-	-	√	-	-	-	√	-
TA05580	IIIb	TP03_0866	0.0314	43.45	61.61	√	-	-	√	-	-	-	√	√
TA09785	IVa	TP04_0920	0.1289	38.63	56.47	-	-	-	√	√	-	-	√	√
TA09790	IVa	TP04_0917	0.0059	37.22	56.33	√	-	-	√	-	-	-	√	√
TA09795	IVa					-	-	-	√	-	-	-	√	√
TA09800	IVa					√	-	-	√	-	-	-	√	√
TA09805	IVa	TP04_0916	0.1982	45.45	60.99	√	-	-	√	-	-	-	√	√
TA09810	IVa					√	-	-	√	√	-	-	√	√
TA09865	IVa					√	-	-	√	√	-	-	√	√
TA17120	IVb	TP04_0013	0.1375	47.16	62.02	-	-	-	√	-	-	-	√	√
TA17125	IVb	TP04_0017	0.2953	50.58	68.42	√	-	-	√	-	-	-	√	√
TA17130	IVb					-	-	-	√	-	-	-	√	√
TA17135	IVb	TP04_0018	0.2753	52.79	70.2	-	-	-	√	-	-	-	√	√
TA17140	IVb	TP04_0019	0.1779	57.91	70.72	-	-	-	-	-	-	-	√	√

Locus = sub-telomeric location (chromosome I-IV / sub-telomere a or b), $d_N d_S$ = ratio of non-synonymous to synonymous substitution rate, SignalP3.0 = signal peptide motif, TMD = transmembrane domain, GPI = GPI anchor, NLS = nuclear localisation signal, FAINT = FAINT domain, PEST = PEST motif, ND = no data. The 4 SVSP members selected for allelic sequencing (SVSP1-4) are highlighted
