# Supplementary material for: Evolution and diversity of secretome genes in the apicomplexan parasite Theileria annulata
Source: BMC Genomics. 2010 Jan 18;11:42. doi: 10.1186/1471-2164-11-42 (PMC2826314; doi:10.1186/1471-2164-11-42)
Supplement: Additional file 3 — Parasite material. The type and origin of parasite material used to prepare template DNA [file 1471-2164-11-42-S3.PDF]

Additional file 3 - Parasite material

Study ID	Type	Origin	MS genotyping (alleles / locus)		Details
			Mean	Max	
t005	piroplasm	Sarikoy village, Aydin, Turkey	5.4	9	Collected 2001
t021	piroplasm	Sarikoy village, Aydin, Turkey	5.6	11	Collected 2001
t029	piroplasm	Sarikoy village, Aydin, Turkey	5.7	10	Collected 2001
t038	piroplasm	Sarikoy village, Aydin, Turkey	6.3	12	Collected 2001
w019	cell line	Northern Tunisia (site 1 *)	1	1	14B *
w027	cell line	Northern Tunisia (site 4 *)	1	1	19 *
w030	cell line	Northern Tunisia (site 4 *)	1	1	22 *
w032	cell line	Northern Tunisia (site 3 *)	1	1	24A (2c) *
w050	cell line	Northern Tunisia (site 13 *)	1	1	42 *
w062	cell line	-	1	1	527 cl 4 *
w067	cell line	-	1	1	29 cl 4 *
w073	cell line	-	1	1	cl 2 BV4 cl5 *
w101	cell line	Northern Tunisia	1	1	Jedeida 4
w102	cell line	Northern Tunisia	1	1	Batan 2

MS = micro- and mini-satellite, * details in Ben Miled *et al.*, Genomic and phenotypic diversity of Tunisian *Theileria annulata* isolates. Parasitology 1994, 108 (Pt 1):51-60.
