# Supplementary material for: Evolution and diversity of secretome genes in the apicomplexan parasite Theileria annulata
Source: BMC Genomics. 2010 Jan 18;11:42. doi: 10.1186/1471-2164-11-42 (PMC2826314; doi:10.1186/1471-2164-11-42)
Supplement: Additional file 4 — Genes selected for allelic sequencing. Details of each of the genes chosen for allelic sequencing including orthologous genes in T. parva, interspecies dNdS values, EST expression data in T. annulata and bioinformatic motif predictions [file 1471-2164-11-42-S4.PDF]

Additional file 4 - Genes selected for allelic sequencing

Gene name	*T. annulata* GeneDB ID	*T. Parva* locus	d_N	d_S	d_Nd_S	nucleotide identity (%)	protein identity (%)	*T. annulata* gene				
								Macro	Mero	Piro	Signal	NLS
SVSP1	TA16025	TP02_0955	0.467	1.157	0.4037	65.60	43.21	√	-	-	√	-
SVSP2	TA17485	TP01_1225	0.477	1.530	0.3118	62.40	44.97	√	√	-	√	-
SVSP3	TA17545	TP04_0002	0.468	2.607	0.1796	45.86	61.07	√	-	-	√	√
SVSP4	TA16045	TP03_0001	0.500	2.391	0.2091	44.25	60.23	√	-	-	√	√
TashHN	TA20090	TP01_0603	0.218	0.822	0.2646	77.11	65.36	√ *	-	-	√	√
SuAT$_1$	TA03135	TP01_0617	0.449	2.048	0.2192	62.93	47.40	√ *	-	-	√	√
TashAT2	TA20095	TP01_0602	0.366	1.432	0.2554	52.80	67.04	√	-	-	√	√
TashAT3	TA20082	-	-	-	-	-	-	√	-	-	√ †	√

d_N = rate of non-synonymous substitutions, d_S = rate of synonymous substitutions, d_Nd_S = ratio of non-synonymous to synonymous substitution rate, Macro, Mero & Piro = macroschizont, merozoite and piroplasm EST data, Signal = signal peptide motif, TMD = transmembrane domain, * experimental data, † SignalP3.0, neural network
