# Supplementary material for: Evolution and diversity of secretome genes in the apicomplexan parasite Theileria annulata
Source: BMC Genomics. 2010 Jan 18;11:42. doi: 10.1186/1471-2164-11-42 (PMC2826314; doi:10.1186/1471-2164-11-42)
Supplement: Additional file 5 — PCR and sequencing primers. Oligonucleotide sequences for the PCR and sequencing primers used in this study [file 1471-2164-11-42-S5.PDF]

Additional file 5 - PCR and sequencing primers

Gene name	PCR primers		Additional sequencing primers (sense & anti-sense)	
	Forward	Reverse	Internal locus 1	Internal locus 2
SVSP1	CATGGGTCAATGTCAAATAACATTC (5' UTR, -38 TO -14)	CATAAACTTACATCATATAG (3' UTR, +40 TO +21)	CAGACCCTCCTTTACCTA TAGGTAAAGGAGGGTCTG	CAGTGTTTCATTGTCTACTAGG CCTAGTAGACAATGAAACACTG
SVSP2	ATGAATAAATACGTTAGATACAC (1 TO 23)	GACGATTCTAAGTTTTATGTGC (3' UTR, +25 TO +4)	CCTCCAGCAATTGAGTAT ATACTCAATTGCTGGAGG	-
SVSP3	CTGTATTATATAACTATGAAATGC (-15 TO 9)	CATCTTTACTACGTCTTCGTCTCG (1246 TO 1223)	CCTAGTCAACTACCACATAC GTATGTGGTAGTTGACTAGG	-
SVSP4	CTATGAAATGCAAAATATATGC (-2 TO 20)	CCATTACAATATATAAACAATGTG (3' UTR, +69 TO +46)	ATATTGAGCCTAGTAAAAC GTTTTACTAGGCTCAATAT	-
TashHN	ATGACCAGATTAAAGATTGC (1 TO 20)	GTGTTCAATTATGGTGGCTTGTG (3' UTR, +28 TO +6)	GACCAACTCATTCAAGAG CTCTTGAATGAGTTGGTC	-
SuAT1	CCTTGTATTGTTTTTACAG (19 TO 37)	GATGATTTGTTTCATGTCTTC (3' UTR, +32 TO +12) * GGTTGTAATTCTAAATGTTCTGG (1487 TO 1465) †	TGAAGAGACGGATACTGC GCAGTATCCGTCTCTTCA	CCTAGGATACGTAGACCT AGGTCTACGTATCCTAGG
TashAT2	TTAGCTGAGGAGGGAATTGATCTAGAGAAG (862 TO 891) *(TASHAT2 SPECIFIC)*	CATCTTCAGCATCTCGATCTTCAC (1582 TO 1559) *(TASHAT2/3)*	-	-
TashAT3	TTAGCTGAGGAAGGAATTGATCTAGAAAAG (565 TO 594) *(TASHAT1/3)*	CATCTTCAGCATCTCGATCTTCAC (1528 TO 1505) *(TASHAT2/3)*	-	-

* used to amplify Tunisian clones, † used to amplify Turkish isolates
